# Concanavalin A promotes angiogenesis and proliferation in endothelial cells through the Akt/ERK/Cyclin D1 axis

**DOI:** 10.1080/13880209.2021.2013259

**Published:** 2021-12-16

**Authors:** Jing-Zhou Li, Xiao-Xia Zhou, Wei-Yin Wu, Hai-Feng Qiang, Guo-Sheng Xiao, Yan Wang, Gang Li

**Affiliations:** Xiamen Cardiovascular Hospital, Xiamen University, Xiamen, Fujian, China

**Keywords:** Cell proliferation, cell cycling, HUVECs, VEGF, bFGF, PDGF

## Abstract

**Context:**

Concanavalin A (Con A) exhibited multiple roles in cancer cells. However, the role of Con A in endothelial cells was not reported.

**Objective:**

Our present study investigated the potential angiogenic role of Con A in endothelial cells and ischaemic hind-limb mice.

**Materials and methods:**

Human umbilical vein endothelial cells and Ea.hy926 cells were employed to determine the effect of Con A (0.3, 1, and 3 μg/mL) or vehicle on angiogenesis and cell proliferation with tube formation, ELISA, flow cytometry, EdU, and western blot. Hind-limb ischaemic mice were conducted to determine the pro-angiogenic effect of Con A (10 mg/kg) for 7 days.

**Results:**

Con A promoted tube formation to about three-fold higher than the control group and increased the secretion of VEGFa, PDGFaa, and bFGF in the medium. The cell viability was promoted to 1.3-fold by Con A 3 μg/mL, and cell cycle progression of G0G1 phase was decreased from 77% in the vehicle group to 70% in Con A 3 μg/mL, G2M was promoted from 15 to 19%, and S-phase was from 7 to 10%. Con A significantly stimulated phosphorylation of Akt and ERK1/2 and expression of cyclin D1 and decreased the expression of p27. These effects of Con A were antagonised by the PI3K inhibitor LY294002 (10 μM) and MEK pathway antagonist PD98059 (10 μM). Moreover, Con A (10 mg/kg) exhibited a repair effect in ischaemic hind-limb mice.

**Discussion and conclusions:**

This study will provide a new option for treating ischaemic disease by local injection with Con A.

## Introduction

The endothelium plays a vital role in vascular homeostasis in the interior surface of blood vessels and lymphatic vessels (Deanfield et al. 2007). Endothelial cells (ECs), which are essential components of the endothelium, release different cytokines and growth factors that mediate cellular adhesion, vessel wall inflammation, and angiogenesis in response to physical and/or chemical signals (Flammer et al. 2012). ECs have immense potential in regenerative medicine, particularly cellular therapeutic strategies aiming to enhance tissue-engineered constructs or rejuvenate ischaemic tissues via neovascularization (Chen et al. 2019). Although several approaches have succeeded in generating ECs proliferation and angiogenesis, such as conditioning medium from adipose-derived stem cells (Luo et al. 2018), fatty acid synthase (Singh et al. 2017), and hypoxia-induced mitogenic factor (Tong et al. 2006), the low efficiencies and limitations involved in them constitute obstacles in massively and safely generating ECs for further applications.

Concanavalin A (Con A), a long-studied representative legume lectin, is extracted from jack beans and identified to bind to the surface of glycoproteins and glycolipids in many cell types, including leukocytes, keratinocytes, hepatocytes, and a large number of cell lines, and was also reported to directly stimulate B cells to synthesize DNA and to proliferate (Pink et al. 1983; McMillan et al. 1984; Peschke et al. 1990; Schaumburg-Lever 1990). Thus far, Con A has been generated rising attention for its anti-proliferative and antitumor activities towards various types of cancer cells. Con A has been reported to kill tumour cells targeting apoptosis, autophagy, anti-angiogenesis, and immunomodulatory (Li et al. 2011). However, the role of Con A on cell cycle progression and angiogenic activity in ECs was barely reported.

In the present study, we will investigate the role of Con A on cell proliferation and angiogenic activity in ECs, and explore the involved signalling pathway. The therapeutic potential of Con A in angiogenesis was determined in ischaemic hind-limb mice. The results showed that Con A significantly promoted endothelial cell proliferation, cell cycling, and angiogenic activity via pAkt, pERK1/2, and cyclin D1 in human umbilical vein endothelial cells (HUVECs) and Ea.Hy926 endothelial cells. Treatment of ischaemic hind-limb mice by local injection with Con A can significantly improve the function and blood flow of ischaemic hind-limb.

## Materials and methods

### Materials

BD Matrigel™ Basement Membrane Matrix, culture dishes, and plates were obtained from Corning Inc. (Corning, NY, USA). Concanavalin A (Con A), sodium dodecyl sulphate (SDS), and bovine serum albumin (BSA) were purchased from Sigma-Aldrich (St. Louis, MO, USA). Endothelial Cell Medium (ECM) was obtained from ScienCell (Carlsbad, CA, USA). PD98059 and LY294002 were the products of Tocris Bioscience (Bristol, UK). The primary antibodies, such as rabbit anti-phospho-Akt (Ser473) (#4060S), rabbit anti-Akt (#9272S), rabbit anti-phospho-ERK1/2 (#4730S), rabbit anti-ERK1/2 (#4695S), rabbit anti-phospho-p38 (#4511S), rabbit anti-p38 (#8690S), mouse anti-p27 (#3686S), mouse anti-p21 (#2947S), rabbit anti-cyclin D1 (#55506S), and rabbit anti-cyclin E (#20808S) antibodies were from Cell Signalling Technology (Beverly, MA, USA). The polyvinylidene fluoride (PVDF) membranes were the products of Millipore (Billerica, MA, USA). EdU Apollo®488 *In Vitro* Imaging Kit was a product of RIBOBIO (Guangzhou, China).

### Cell culture

HUVECs were isolated from the umbilical vein of the umbilical cord of newborns from Zhongshan Hospital of Xiamen University, followed by the ethnic committee of Xiamen University, and the consent form was also received from patients. The protocol of isolation HUVECs was described as previous (Baudin et al. 2007). Briefly, HUVECs were isolated from the umbilical vein vascular wall by a collagenase treatment, then seeded on fibronectin-coated plates and cultured in a medium with Earles’ salts and foetal calf serum (FCS), but without growth factor supplementation, for 7 days in a 37 °C–5% CO_2_ incubator. Cell confluency can be monitored by phase-contrast microscopy; HUVECs can be characterized using cell surface or intracellular markers and checked for contamination. EA.hy926 cells were bought from ATCC (Manassas, VA, USA). HUVECs were maintained in Endothelial Cell Medium (ECM), supplemented with 5% foetal bovine serum (FBS), 1% Endothelial Cell Growth Supplement, 100 U/mL penicillin, and 100 μg/mL streptomycin in a humidified atmosphere of 5% CO_2_ at 37 °C. EA.hy926 cells were maintained in Dulbecco’s Modified Eagle Medium (DMEM) supplemented with 10% foetal bovine serum (FBS), 100 U/mL penicillin, and 100 μg/mL streptomycin. After treatment with Con A (0.1, 0.3, 1, 3 μg/mL) in FBS-free medium for 24 h in HUVECs and EA.hy926 cells, cells were detected with different methods, such as MTT assay, EdU assay, cell cycle by flow cytometry assay, Matrigel tube formation, ELISA, and Western blot assay. For examining the signalling pathway, after pre-treated with PD98059 (10 μM) or LY294002 (10 μM) for 1 h, the HUVECs were then treated with 3 μg/mL Con A for an additional 24 h.

### Matrigel tube formation assay

The 96-well cell culture plates were pre-coated with 50 µL growth-factor–reduced Matrigel™. HUVECs or EA.hy926 cells (2.0 × 10^4^, serum-starved for 24 h) were mixed with FBS-free medium (negative control), or 5% FBS medium (positive control), or Con A (0.3, 1, 3 μg/mL) in FBS-free medium, and then seeded in the pre-coated plates. Six hours later, the tube formation ability of HUVECs was captured by microscopy. Total master tube length was calculated using the Image J software plug-in angiogenesis module (National Institutes of Health, USA).

### ELISA assay for angiogenesis-related cytokines

Cells were cultured in 6-well plates and treated with different concentrations of Con A (0.3, 1, 3 μg/mL) for 24 h; the supernatant of the culture medium was collected for ELISA assay. The concentration of VEGF, bFGF, and PDGFaa in the supernatant was quantified using the human VEGF, bFGF, or PDGFaa ELISA Ready-SET-GO kit (eBioscience, San Diego, CA, USA) according to the manufacturer’s instructions.

### MTT assay

The viability of treatment with Con A on HUVECs was performed using the 3-(4, 5- dimethyl-2-thiazolyl)-2, 5-diphenyl-2H-tetrazolium bromide (MTT) assay. Cells were seeded into 96-well plates at a density of 5–8 × 10^3^ cells/well in 100 μL medium. Cells were treated with different concentration of Con A (0.1, 0.3, 1, 3 μg/mL) in 100 μL medium with 1% FBS for 24 h. The MTT solution 10 μL (5 mg/mL) was treated in each well at 37 °C for 4 h. Formazan crystals were dissolved with 150 μL Dimethyl sulfoxide (DMSO) after removing the medium. The optical density values were read using a microplate reader (TECAN infinite M200 pro, USA).

### EdU incorporation assay and confocal microscopy

Approximately 2.5 × 10^5^ cells were seeded in 6-well culture plates with a coverslip in each well for EdU (5-ethynyl-2′-deoxyuridine) incorporation assay. After pretreating with antagonists for 1 h or/and treatment with Con A (0.1, 0.3, 1, 3 μg/mL) for an additional 24 h, cells were incubated with EdU for an additional 1 h followed by fixation with 4% paraformaldehyde. Cells were then incubated with rabbit anti-EdU primary antibody overnight at 4 °C. After washing with PBS 3 times, Alexa 488-conjugated goat anti-rabbit secondary antibodies were used for 1 h. Cells were counterstained with 6-diamidino-2-phenylindole (DAPI) for nuclear staining. Images were captured using Leica SP5-II laser scanning confocal microscopy (Leica, Germany). Image J software (NIH, USA) was applied to calculate the percentage of EdU-positive cells.

### Cell cycle analysis

Cell cycle distribution was detected using flow cytometry in HUVECs as described previously (Che et al. 2015; Zhang et al. 2015; Li et al. 2017, 2018). After pre-treated antagonists for 1 h and incubated with different concentrations of con A (0.1, 0.3, 1, 3 μg/mL) for an additional 24 h, cells were harvested by 0.25% trypsin for flow cytometry analysis. Cells were washed with PBS 3 times and fixed in ice-cold 70% ethanol overnight at 4 °C. Then the cells were washed and incubated with 0.2 mL staining solution (0.1% Triton-X 100 in PBS with 20 μg/mL propidium iodide and 10 μg/mL RNase A) for 30 min. Cell cycle distributions were performed by fluorescence-activated cell sorting using a Beckman Coulter flow cytometry (Gallios). The G0/G1, S, and G2/M phase cell percentages were calculated with MODFIT LT software (BD Biosciences, USA).

### Limb ischaemia mice

Limb ischaemia was conducted in C57/BL6 mice purchased from Vital River Laboratory Animal Technology Company (Beijing, China). Animal care and experimental procedures were performed following instructions of the Institutional Animal Care and the Ethics Committee for animal experiments at Xiamen University (Approval code: XMULAC20170004). A total of 18 male mice were equally assigned to the following 3 groups: Sham group (*n* = 6), vehicle group (injected with saline solution) (*n* = 6), and the Con A (10 mg/kg) treatment group (*n* = 6). In the vehicle and Con A group, the femoral artery of the left hind was excised from its proximal origin as a branch of the external iliac artery to the distal point where it bifurcates into the saphenous popliteal arteries. Then Con A (10 mg/kg) or an equal volume of vehicle was injected into the tissue at 4–5 points around the femoral artery. Tissue perfusion of the hind limbs was assessed with a laser Doppler perfusion imager (Perimed, Stockholm, Sweden). The digital colour-coded images were analyzed to quantify blood flow in the stripped area of the blood vessel.

### Western blot analysis

After different treatments, cells were treated with RIPA lysis buffer according to the manufacturer’s instructions (Beyotime, Shanghai, China). Proteins were separated by 10 or 12% polyacrylamide gel electrophoresis and transferred to polyvinylidene difluoride (PVDF) membranes. The membranes were then incubated in blocking buffer (5% non-fat milk, 0.1% Tween 20 in Tris-buffer saline buffer) for 1 h and then incubated overnight at 4 °C with different primary antibodies in dilution factors from 1:500 to 1:2000. After that, the membranes were incubated with secondary antibodies coupled with horseradish peroxidase (1:5000 − 1:10,000) for 1 h at room temperature. After being washed three times, the bound antibody was detected by the Western Lightning ECL reagents by the chemiluminescence imaging system. The relative band intensities of the blots were calculated by image analysis software Image J.

### Statistical data analysis

All the data are representative of five or more independent experiments. Data were shown as mean ± S.E.M. Statistical significance was assessed using Graphpad prism 5.0 with a one-way ANOVA analysis followed by Tukey’s test for multiple group tests. *p* Values of less than 0.05 were considered to indicate statistical significance.

## Results

### Effects of Con A on angiogenic activity in HUVECs

**Figure 1.**
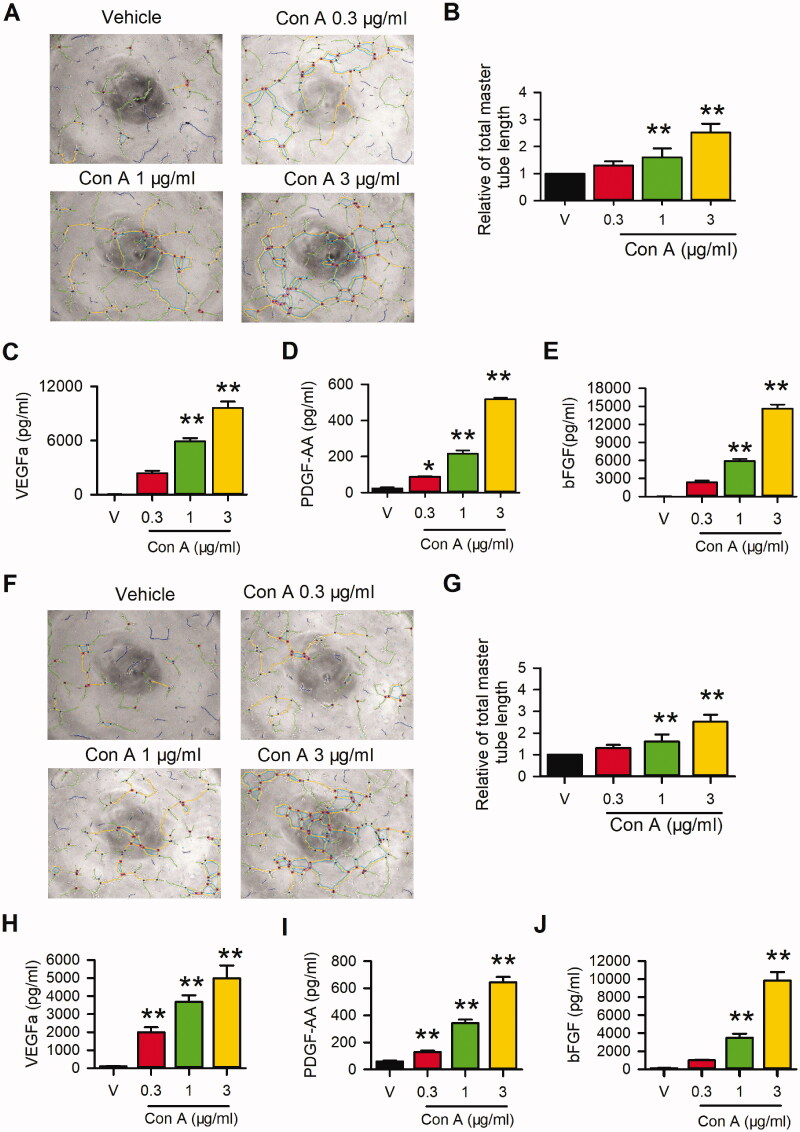
Effects of Con A on angiogenic activity in HUVECs and EA.hy926 cells. Images of Matrigel tube formation of Con A (0.3, 1, 3 μg/mL, 6 h)-treated HUVECs (A) and EA.hy926 cells (F), and analysis of total master segments length in HUVECs (B) or EA.hy 926 (G) (*n* = 6). ELISA assay showed the concentrations of VEGFa, PDGFaa, and bFGF, respectively, in the supernatant of HUVECs (C, D, E) and EA.hy926 (H, I, J) (*n* = 5) (*p < 0.05, **p < 0.01 vs. vehicle).

The pro-angiogenic activity of Con A was explored in HUVECs and EA.hy926 cells using Matrigel tube formation and ELISA assay. As shown in Figure 1, different concentrations of Con A, especially at 1 or 3 μg/mL, significantly promoted the tube formation in HUVECs (Figure 1(A)) and Ea.hy926 cells (Figure 1(F)). The total master segment length in HUVECs (Figure 1(B)) and EA.hy926 cells (Figure 1(G)) showed significantly elevated in treatment with Con A at 1 or 3 μg/mL treated cells compared to the negative control.

To determine whether the angiogenesis-related cytokines are involved in the pro-angiogenic effect of Con A in HUVECs and Ea.hy 926, we detected the concentration of VEGF, PDGFaa, and bFGF in the medium from Con A-treated HUVECs. The results showed that Con A significantly increased the concentration of VEGFa, PDGFaa, and bFGF in the medium from HUVECs (Figure 1(C–E)) or Eahy 926 (Figure 1(H–J)). These results indicated that Con A promoted angiogenic activity by increasing the production of VEGF, PDGFaa and bFGF secreted from endothelial cells.

### Effects of Con A on cell proliferation and cell cycling progression in HUVECs

**Figure 2.**
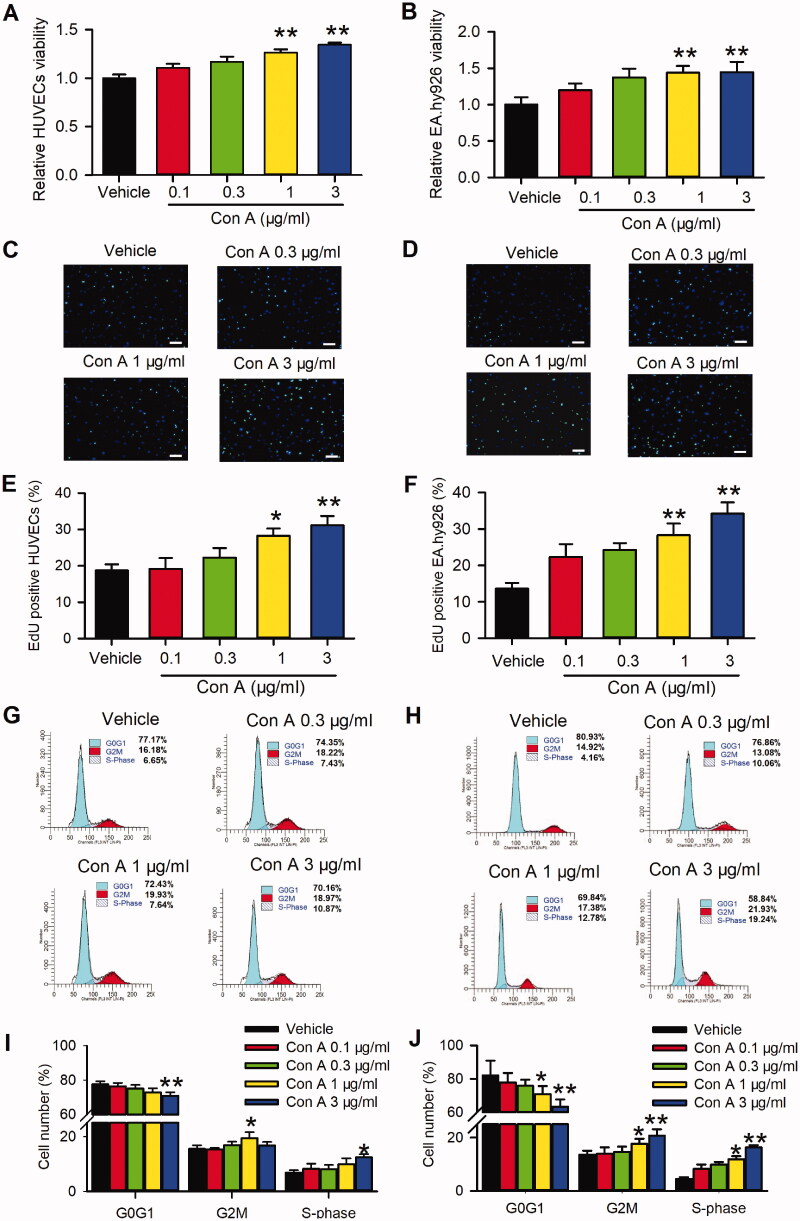
Effects of Con A on cell proliferation in HUVECs and EA.hy926 cells. Relative of cell viability in HUVECs (A) and EA.hy926 cell (B) treated with vehicle (PBS) or Con A (0.1, 0.3, 1, 3 μg/mL) using MTT assay. Immunofluorescent staining graphs of EdU in HUVECs (C) or Ea.hy926 cells (D) treated with vehicle (PBS) or Con A (0.3, 1, 3 μg/mL). Percentage values of EdU positive nuclei in HUVECs (E) or EA.hy926 cells (F) treated with vehicle (PBS) or different concentrations of Con A (0.1, 0.3, 1, 3 μg/mL). Flow cytometry graphs showing the distribution of cell cycle phases treated with vehicle or Con A (0.3, 1, 3 μg/mL) in HUVECs (G) or EA.hy926 cells (H). Percentage values of cell cycling population at different phases in HUVECs (I) or EA.hy926 cells (J) treated with Con A (0.1, 0.3, 1, 3 μg/mL) for 24 h (*n* = 5, *p <0.05, **p <0.01 vs. vehicle).

To our knowledge, the role of pro-proliferation in vascular endothelial cells is essential for angiogenesis. Hence, we determined cell proliferation in con A-treated HUVECs using MTT assay, EdU incorporation assay, and cell cycle detection with flow cytometry. As shown in Figure 2(A,B), the MTT assay represented Con A significantly promoted cell viability. The pro-proliferation effect of Con A on HUVECs was validated by EdU incorporation assay in HUVECs (Figure 2(C,D)) and EA.Hy926 (Figure 2(E,F)). The results showed more EdU positive cells in the presence of Con A 1 and 3 μg/mL than vehicle control (Figure 2(C,E)). Figure 2(D,F) expressed the EdU positive nuclei cell number statistics, in which positive cells were significantly promoted from about 15–30% after being treated with Con A 1 or 3 μg/mL.

Cell cycle progression plays an essential role in the cell proliferation process. We then explored the effect of Con A on the cell cycle with flow cytometry in HUVECs. Figure 2(G) (HUVECs) and 2H (EA.hy926) showed the representative of the cell cycle distribution with flow cytometry graphs. Figure 2(I,J) illustrate the percentage of cycling progression phases in HUVECs (Figure 2(I)) or EA.hy926 cells (Figure 2(J)) treated with vehicles or different concentrations of Con A. In Con A-treated cells, especially 3 μg/mL, the cells in the G0/G1 phase were significantly decreased, while the cells in S-phase and G2/M phase were increased considerably. These results suggest that Con A-promoted angiogenic effect in HUVECs may mainly be mediated by cell proliferation via promoting the G0/G1 boundary to the S phase.

### Effect of Con A in hind-limb ischaemia mice

**Figure 3.**
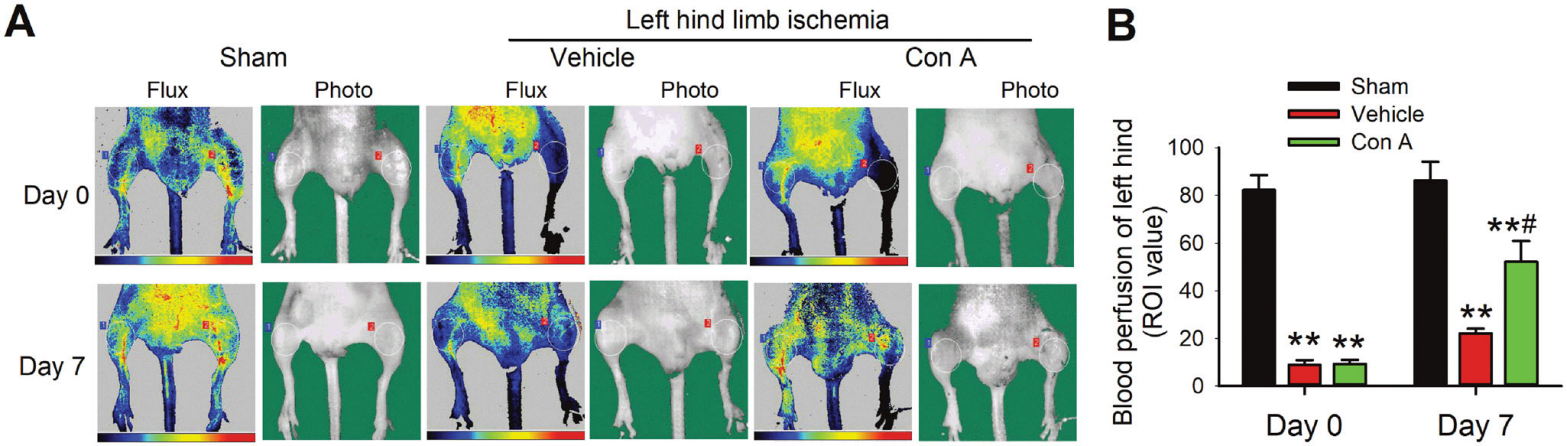
Improvement of blood flow and limb functions on ischaemia hindlimb in the treatment with Con A. (A). Representative laser Doppler flow imaging showed dynamic changes in blood perfusion in limb ischaemia at days 0 and 7. (B). The blood perfusion with the region of interest (ROI) value was tested by laser Doppler flow in three groups (*n* = 6, *p <0.05, **p <0.01 vs. vehicle).

Hind-limb ischaemia in mice is an important animal model for investigating the angiogenesis effect of drugs *in vivo*. To determine the angiogenic potential of Con A in ischaemic tissue, we locally injected Con A into the hind-limb ischaemia model of mice. After 7 days of ischaemia, the blood perfusion in the vehicle group was significantly decreased, while treatment of Con A improved the blood perfusion (Figure 3(A,B)), which confirmed the viewpoint that Con A exhibited a strong pro-angiogenic effect.

### Signal molecules involved in Con A regulated cell cycling progression and angiogenic activity

**Figure 4.**
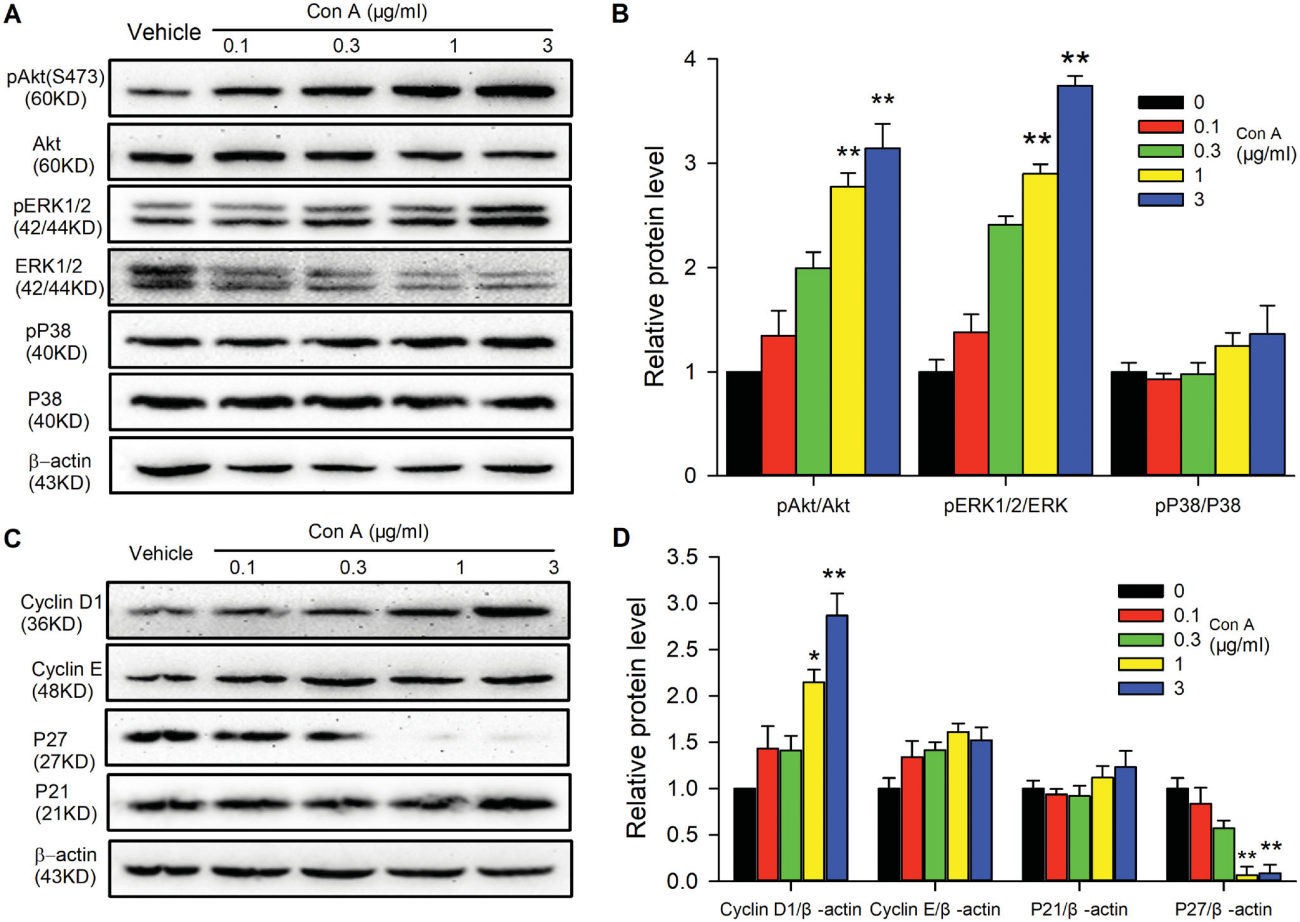
Molecular signals in Con A-regulated proliferation and angiogenesis in HUVECs. (A). Representative images of Western blot of pAkt (Ser473), Akt, pERK1/2, ERK1/2, pP38, and P38 in HUVECs treated with Con A. (B). Relative pAkt, pERK1/2, and pP38 expression in HUVECs treated with Con A (*n* = 5). (C). Represent images of western blot of cyclin D1, cyclin E, P27, and P21 in HUVECs treated with Con A. (D). Relative values of cyclin D1, cyclin E, P27, and P21 in HUVECs treated with Con A (*n* = 5, *p < 0.05, **p < 0.01 vs. vehicle).

To determine the signal molecules involved in Con A-regulated cell proliferation and angiogenic activity, the proliferation-related molecules were determined with western blot in HUVECs in the absence or presence of different concentrations of Con A. Figure 4 illustrates the effect of Con A on survival kinase Akt, the mitogen-activated protein kinase P42/44 (ERK1/2), P38, and the cell cycle-related proteins cyclin D1, cyclin E, P21, and P27 in HUVECs. It is interesting to note that phosphorylation of Akt (Ser473) (Figures 3(B) and 4(A)) and ERK1/2 (Figure 4(A,B)) and the expression of cyclin D1 (Figure 4(C,D)) were significantly increased by Con A. In contrast, the expression of p27 was decreased considerably. However, the expression of cyclin E (Figure 4(C,D)), P38 (Figure 4(A,B)), and P21 (Figure 4(C,D)) did not have any significant statistical differences.

### PI3K and MEK pathway participated in Con A-regulated cell proliferation and angiogenesis in HUVECs

**Figure 5.**
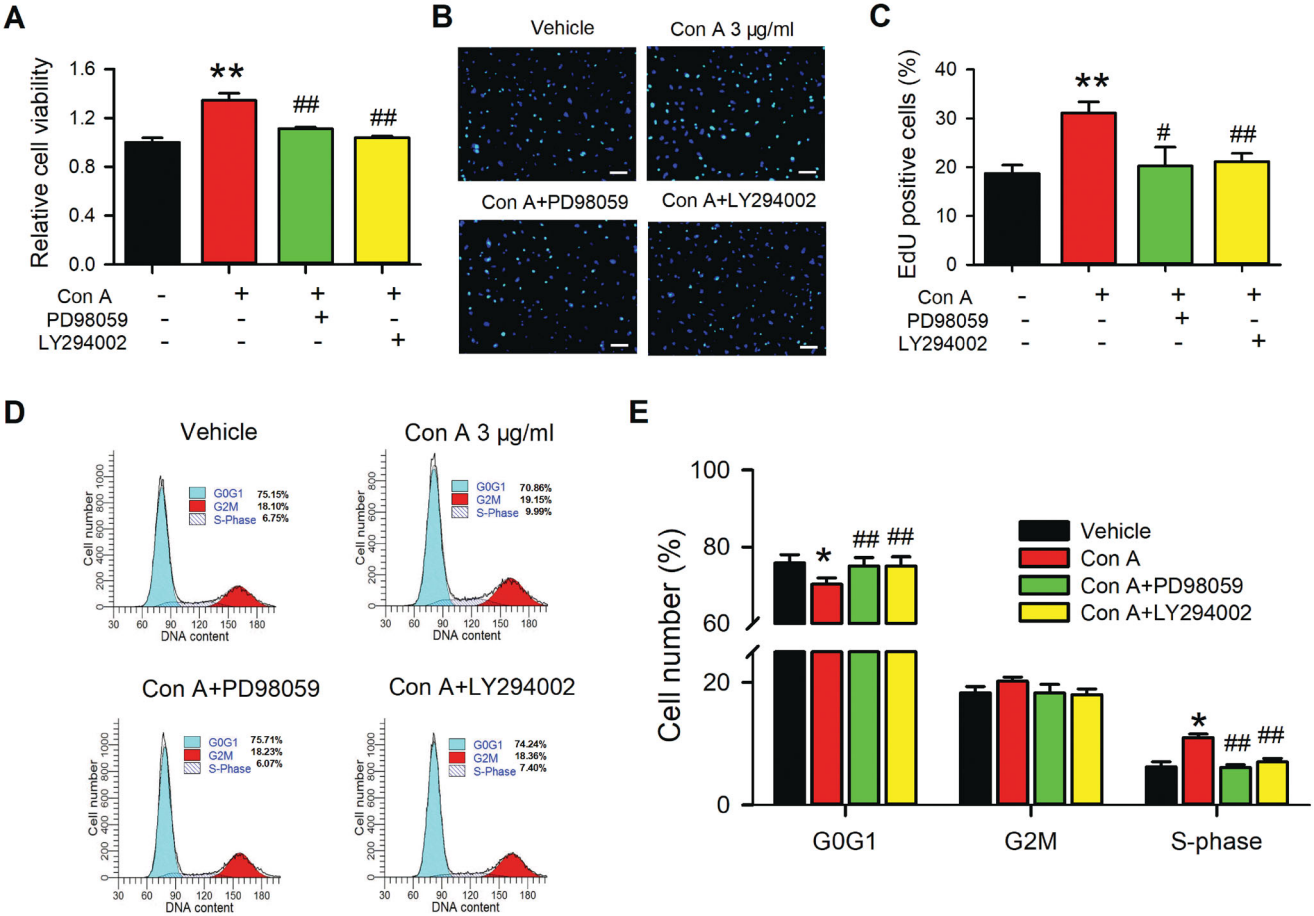
Effects of antagonists of the signal pathway on Con A-induced cell proliferation in HUVECs. (A). Relative of cell viability in HUVECs treated with vehicle (PBS), Con A (3 μg/mL), or Con A (3 μg/mL) plus with PI3K blocker LY294002 (10 μM) or MEK inhibitor PD98059 (10 μM) determined with MTT assay. Representative images (B) and Percentage values (C) of immunofluorescent staining of EdU in HUVECs treated with vehicle (PBS), Con A (3 μg/mL), or Con A (3 μg/mL) plus with LY294002 (10 μM) or PD98059 (10 μM). Representative images (D) and Percentage values (E) of flow cytometry graphs showing the distribution of cell cycle phases treated with vehicle (PBS), Con A (3 μg/mL), or Con A (3 μg/mL) plus with LY294002 (10 μM) or PD98059 (10 μM) (*n* = 6, *p <0.05, **p <0.01 vs. vehicle; ^#^*p* <0.05, ^##^*p* <0.01 vs. Con A).

It has been reported that PI3K and MEK kinase mediate the activation of pAkt, pERK1/2, and/or cyclin D (Blaes and Girolami 2013; Nicoletti et al. 2014; Fu et al. 2015; Yu et al. 2016). To further investigate whether the PI3K and MEK kinase are involved in Con A-mediated cell proliferation and angiogenic effect in HUVECs, we utilized the PI3K antagonist LY294002 and the MEK kinase inhibitor PD98059 on the Con A-treated HUVECs using MTT, EdU assay, flow cytometry, and western blot (Figure 5). The MTT assay showed that the PI3K antagonist LY294002 and the MEK kinase PD98059 significantly decreased Con A-induced cell viability promotion (Figure 5(A)). Moreover, the proliferation induced by Con A in HUVECs was also reduced by LY294002 and PD98059 (Figure 5(B,C)). Con A-induced cell cycle transition was also restrained by the two inhibitors (Figure 5(D,E)).

**Figure 6.**
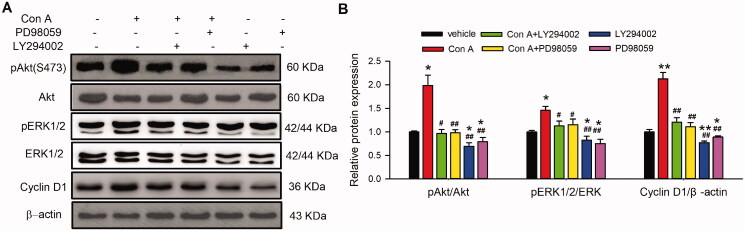
Effect of PI3K and MEK on Con A-induced cell proliferation-related molecular signal pathway. Images (A) and relative values (B) of western blots of pAkt (Ser473), Akt, pERK1/2, ERK1/2, cyclin D1 in cells treated with Con A (3 μg/mL) or Con A (3 μg/mL) plus with LY294002 (10 μM) or PD98059 (10 μM) (*n* = 5, *p <0.05, **p <0.01 vs. vehicle; ^#^*p* <0.05, ^##^*p* <0.01 vs. Con A).

The role of PI3K and MEK kinase antagonists in Con A-regulated phosphorylation of Akt and ERK and expression of cyclin D1 and P27 were also explored. Interestingly, LY294002, but not PD98059, significantly reduced Con A-promoted the phosphorylation of pAkt. However, Con A-increased pERK1/2 and cyclin D1 were decreased by LY294002 and PD98059 (Figure 6(A,B)). These results suggest that PI3K and MEK are involved in Con A-induced proliferation and angiogenesis via Akt/ERK1/2/cyclin D1 pathway.

## Discussion

During atherosclerosis, endothelial cells injury or apoptosis was induced by numerous risk factors such as smoking, hyperlipidaemia, diabetes, and vascular inflammation (Chen et al. 2000; Pober et al. 2009). Endothelial cell (ECs) injury is an essential deleterious factor for endothelial integrity, while the proliferated and healthy ECs can sustain endothelial integrity (Schober et al. 2014). Concanavalin A (Con A) is a plant lectin extracted from jack beans and binds specifically to terminal mannose or glucose residues on cell surface glycoconjugates, such as glycoproteins, regulates physiological functions (Sasaki and Toyoda 2013). Con A has shown the effects of stimulating cell immunity and generating an immune memory, reported as a phytohemagglutinin with potent mitogenic effects (Li et al. 2011), and is associated with a variety of biological effects, such as mitogenic, cytotoxic, hepatotoxic, and teratogenic (Ballerstadt et al. 2006). However, little is known about the functional role and mechanisms involved in replicative endothelial regeneration of Con A, especially in cell proliferation and angiogenic activity.

Our present study demonstrated that Con A contributes to the human vein endothelial cell proliferation, cell cycle progression, angiogenic activity, and the potential role of Con A in ischaemic tissue repairment. In our results, Con A, with a lower concentration range of 1–3 μg/mL, can significantly promote the HUVECs angiogenesis, proliferation, and cell cycling progression by promoting G0/G1 boundary cells to the S phase. Previous research has demonstrated Con A induces inflammation through elevating the levels of TNF-α, adhesion molecules, transforming growth factor-β1 (TGF-β1), mitogen-activated protein kinases (MAPKs), and signal transducer and activator of transcription 3 (STAT3) in hepatic tissue (Sang et al. 2017; Zhang et al. 2020). Here, our present investigation showed, in HUVECs, Con A promotes phosphorylation of Akt and ERK, which can be suppressed by the treatment of the PI3K antagonist LY294002 and MEK inhibitor PD98059, indicating that Con A-induced cell cycling progression and angiogenesis in HUVECs is mediated by PI3K and MAPK. Our findings are consistent with Gong et al. (2015) that Con A promoted phosphorylation of ERK1/2 and Akt in primary human hepatic sinusoidal endothelial cells.

Con A is previously recognized as an activator for T-lymphocytes, known as effector cells, and plays a remarkable role in the immuno-stimulatory process in allograft rejection, viral infection, or autoimmune diseases in mammals (Gantner et al. 1995). Recent perspectives believed Con A-induced immune responses depend on various cells such as natural killer T (NKT) cells, CD4^+^ T cells, neutrophils, and intrahepatic macrophages, namely, Kupffer cells (KCs) (Meng et al. 2017). Therefore, Con A-activation of T-cell led to cytokine-induced hepatic injury and was most commonly utilized as a model for immune-related hepatitis or as an insulin receptors agonist and, in general T-cell biology (Arsenijevic et al. 2021; Elshal et al. 2021; Ibrahim et al. 2021; Kar et al. 2021; Khan et al. 2021; Li, Gong, et al. 2021; Li, Kong, et al. 2021). Con A stimulates T-cell causing a release of several cytokines such as tumour necrosis factor-α (TNF-α), interferon-gamma (IFN-γ), granulocyte macrophage-colony stimulating factor (GM-CSF), and interleukins (ILs), that maintain inflammatory and immuno-stimulatory processes and may arouse acute toxicity (Miethke et al. 1992). However, barely any evidence has shown the effect of Con A on angiogenesis-related factors secretion in human vascular endothelial cells. Our present result has demonstrated that Con A can significantly promote the pro-angiogenesis factors, such as PDGFaa, VEGF, and bFGF production in HUVECs, which may by participated in the angiogenic effect of Con A in HUVECs.

The pro-angiogenic or anti-angiogenic effect of Con A has previously been shown in various cancer cells. In U87 glioblastoma cells, Con A induces expression of membrane-associated MT1-MMP, which functions in PGE2-induced angiogenesis, activation of pro-MMP-2, and cell death/survival regulation/survival bio-switch activating IKKa and IκB-NF-κB complexes (Sina et al. 2010). Aside from IKK-NF-jB-COX-2 signalling, Con A was also reported triggering the secretion of MMP-9 as inactive zymogens, activating the proteolysis of MMP-9 via PI3K-Akt and SHP-2-MEK-1-ERK signalling in U87 glioblastoma cells (Biswas et al. 2006). Con A blocks cell survival and as a potential anti-neoplastic agent that targets the apoptotic autophagic and anti-angiogenic pathways through SHP-2-Ras-ERK signalling in fibroblasts as well (Ruhul Amin et al. 2003). However, in human vascular endothelial cells, in our present results, we found a piece of novel information that Con A exhibited as a pro-angiogenesis agent by elevation PDGF, bFGF, VEGFa secretion, and promoting ECs proliferation. Moreover, the effect of Con A on promoting cell proliferation and angiogenesis is validated in mice with hind-limb ischaemia. Although pro-angiogenesis and pro-proliferation effect of Con A on ECs, in cancer cells, many previous studies have shown the opposite opinion that Con A induces cancer cell death. Li et al. reviewed that Con A is a potential anti-neoplastic agent targeting apoptosis, autophagy, and anti-angiogenesis for cancer therapy by targeting apoptosis, autophagy, and angiogenesis with a very high concentration (Li et al. 2011; Velasquez et al. 2013). A specific concentration of Con A initiated apoptosis through cross-linking of Con A receptors on cortical neurons (Kulkarni and McCulloch 1995). Several experiments in cancer cells confirmed that Con A-induced apoptotic cell death via a mitochondrial pathway in diverse types of cells, including PU-1.8 cells, human melanoma A375 cells, and human hepatocellular liver carcinoma HepG2 cells (Liu et al. 2009, 2010; Li et al. 2010, 2011). These controversial points of view on Con A indicate Con A may exert a different effect in different cells.

Collectively, the present study demonstrates the novel information that Con A exhibited mitogenic and angiogenic effects in HUVECs via activating Akt/ERK1/2/p27/cyclin D1 pathway, and this pro-angiogenic effect can be utilized in the repairment of ischaemic hindlimb. However, the targeting receptor involved in the Con A-mediated cell proliferation and angiogenesis in HUVECs, and whether the effect of Con A participates in anti-atherosclerosis or the repair of the myocardium in acute myocardial infarction remains to be further elucidated in the future.
